# Geosynthetic Reinforcement of Sand-Mat Layer above Soft Ground

**DOI:** 10.3390/ma6115314

**Published:** 2013-11-19

**Authors:** Jong-Beom Park, Hyun-Soo Park, Daehyeon Kim

**Affiliations:** 1 Shinmyeong Construction Engineering Co., Ltd.; Suncheon 540-080, Korea; E-Mail: bankgeo@hanmail.net; 2 New Millennium Construction Co., Ltd.; Gwangju 500-862, Korea; E-Mail: gphsssj@gmail.com; 3 Department of Civil Engineering, Chosun University, Gwangju 501-759, Korea

**Keywords:** sand-mat system, geosynthetics reinforced, bearing capacity, soft ground

## Abstract

In order to improve the bearing capacity of soft ground for the purpose of getting trafficability of construction vehicles, the reinforcement of geosynthetics for sand-mat layers on soft ground has often been used. As the strength of the geosynthetics increases, and the sand-mat system becomes stronger, the bearing capacity of sand-mat systems will be increased. The depths of geosynthetics, reinforced in sand-mat layers, were varied with respect to the width of footing. The tensile strengths of geosynthetics were also varied to evaluate the effect of reinforcement on the bearing capacity of soft ground. The dispersion angles, with varying sand-mat thicknesses, were also determined in consideration of the tensile strength of geosynthetics and the depths of reinforcement installations. The bearing capacity ratios, with the variation of footing width and reinforced embedment depth, were determined for the geosynthetics-only, reinforced soft ground, 1-layer sand-mat system and 2-layer sand-mat system against the non-reinforced soft ground. From the test results of various models, a principle that better explains the concept of geosynthetic reinforcement has been found. On the basis of this principle, a new bearing capacity equation for practical use in the design of geosynthetically reinforced soft ground has been proposed by modifying Yamanouchi’s equation.

## 1. Introduction

There has been a great deal of progress in the research on the bearing capacity of sand-mats with 1-layer structures at the time of designing the sand-mat method, which is the most common method applied to surface layer texturing for soft ground thus far. In actuality, a 2-layer sand-mat method is also used, for which 2-layer geosynthetics are used for the reduction of the thickness of the sand layer, or for reinforcement to secure the trafficability of moving equipment.

Yamanouchi [1] reported a practical equation, claiming that embankment done after laying geosynthetics supported the load of embankments with the bending rigidity of plate and subgrade reaction, taking the geosynthetics and surrounding soils as plates with hardness. Hirao *et al*. [2,3] measured the effects of synthetic geotextiles to improve the bearing capacity of soft clay ground, using small-scale laboratory model footing tests. They claimed that an important role of bearing capacity was to increase bending rigidity, based on the confining effect of geotextiles by sand particles.

Shin *et al*. [4] carried out a field load test of geogrid-reinforced ground for the reclamation site of Incheon Airport. They reported that the size of the angle for load dispersion (α) d was reduced, depending on the increase of the applied load by unit area of loading plate, while the size of the angle for load dispersion (α) was increased as the number of layers of reinforcement was increased.

Yasuhara *et al*. [5] conducted a laboratory experiment to prove the effects of sand-mat on the geosynthetics, which were used as reinforcement for soft ground. They pointed out that the effect of stiffness increase is the same as the improved effect obtained by applying tensile strength at the end of geogrid geosynthetics.

Ju *et al*. [6,7] claimed that the effect was affected by the width of loading plates and the thickness of sand-mats in the Soft-ground-Geosynthetics-Sand-mat System, and they obtained a large dispersion angle, in the range of 40°–50°, especially in the case of a loading plate, 20 cm thick.

Ju *et al*. [8] carried out a laboratory model experiment to prove the applicability of EPS (Expandable Polystyrene) and the effect of bearing-capacity increase in the surface layer treatment method, which uses geosynthetics and sand-mat. The result of their experiment revealed that, the bearing capacity for the case where a sand-mat was laid for 10 cm was increased by more than, approximately, 2.5 times of that of the ground on which sand-mat was not laid.

Yang *et al*. [9] applied geosynthetics on the surface layer and investigated the effectiveness of geosynthetics. Park *et al*. [10] analyzed the damage of geosynthetics after the application of geosynthetics, and evaluated the correlations between tensile strength and stitching strength, together with the relationships among tension, rupture, piercing, and tearing strength.

Ham *et al*. [11,12] carried out an experimental research to evaluate the bearing-capacity improvement effects of surface layer reinforcement material for soft ground, and proposed a bearing capacity calculation method for super-soft ground using surface layer reinforcement materials.

Ju *et al*. [13] claimed, in the experimental research on the effect of the sand-mat method using sandy ground, that the net bearing capacity of sand-mat was increased as the tensile strength of geosynthetics and the thickness of sand layer, which were the design factors of the sand-mat, were increased. They insisted that the reason for the increase was because the relative density of the sand-mat is relatively larger than that of lower part ground and, therefore, increase in the thickness of sand-mat is closely related to the effect of increase on bearing capacity, and, in addition, because the size of tensile strength induced to geosysthetics was increased. On the other hand, Ahn [14] studied surface-layer reinforcement material to secure trafficability of super-soft ground, and the behavior of ground friction.

Jun *et al*. [15] conducted a centrifugal model test at 50 g gravity level to evaluate the bearing capacity of super-soft ground reinforced with the geosynthetics, and sand layer where trafficability should be secured. Based on the test results, they observed that the bearing capacity was increased with increasing the strength of ground, and also observed the trend of intrusion or local shear failure. As a result, they proposed a method to evaluate bearing capacity to secure the trafficability of equipment by calculating the equation of regression analysis for bearing capacity and settlement quantity depending on shear strength.

This research focused on securing the trafficability of equipment, based on efficient design for sand layers for the 1-layer and 2-layer structured sand-mat method, and the increase of bearing capacity. That is to say, a model experiment was conducted on a 2-layer structured sand-mat system, in which geosynthetics were laid in between the original ground and the sand-mat layer for the increase of bearing capacity, while additionally laying geosynthetics within the sand-mat layer for the prevention of partial settlement and the increase in the stability of shear failure. The ultimate objective of this research is to propose an equation for the bearing capacity of sand-mat systems in order to reduce the thickness of the sand layer and to secure the trafficability for equipment in the design of sand layer on soft-ground, based on the result of the model experiment.

## 2. Laboratory Model Experiment

### 2.1. Laboratory Model Experiment Apparatus

**Figure 1:**
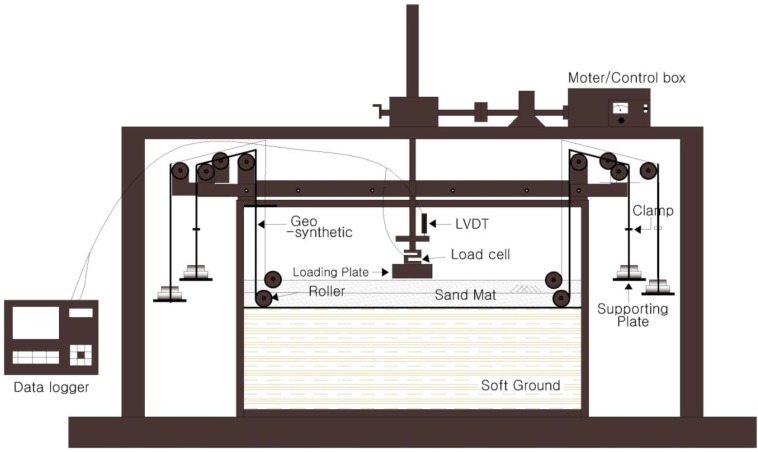
Model loading-experiment apparatus.

The model experiment apparatus used in this study can be categorized into model soil tank, loading plate, loading device, multi-body free falling apparatus, and data logger, as shown in Figure 1, and it is the apparatus which enables the carrying out of an experiment in plane strain condition. Soil tank was manufactured with a width of 120 cm, length of 30 cm, and maximum height of 70 cm, using transparent acrylic plate with a thickness of 2 cm for both side-walls of the soil tank, and silicon grease was used to eliminate the frictional resistance to be generated from the side walls during the experiment. A device, which can artificially add tensile strength to geosynthetics, was attached to the geosynthetics so that the experiment could be carried out under the condition that a prescribed level of tensile strength was applied to the geosynthetics in advance. For the experiment, the loading plate with a size of 10 cm × 29 cm (Length by breadth) was used. Load and settlement were measured simultaneously, and a displacement rate was applied at approximately 1 mm/min. As shown in Figure 1, we used two independent rollers to apply equal tension force for the two reinforcing layers.

Dredging reclamation soil, collected from Yulchon Industrial Complex site in Yeosu City, was used as material when making the model ground. In order to ensure to create the same experimental conditions for the model experiment each time, soft ground with the same conditions should be created. Dredging reclamation soil was used to produce the model ground. For the uniform model ground, the samples were stirred after adding water (at 100% water content), and they were turned into a slurry state. Then, artificially produced samples were put into the model soil tank to make soft ground, with the height of 40 cm, within the model soil tank, and then the loading test was carried out, pursuant to the experiment conditions, after going through a stabilization process for approximately three days. Prior to the loading test, the same water content was maintained by covering the soft ground with vinyl. In all tests, we followed the same procedure for preparing the soft ground.

Woven fabrics with 50 kN/m were used as reinforcement material for geosynthetics, and standard sand from Jumunjin was used for sand-mat. The specific gravity, liquid limit (%), plastic limit (%), Plastic Index (%), and percent passing No. 200 sieve of dredging clay were 2.69, 39.2, 17.0, 22.2, and 98, respectively. The soil used in the study was classified as CL with Unified Soil Classification System (USCS). The weight (g/m^2^), specific gravity, tensile strength (kN/m), tensile elongation (%), and permeability (cm/s) of the polyester woven fabrics were 150, 1.36, 50, 10–30, and 0.0001, respectively. The specific gravity, maximum dry density (kN/m^3^), minimum dry density (kN/m^3^), and relative density (%) of Jumunjin sand were 2.67, 16.54, 13.98, 90, respectively. The sand was classified as SP.

### 2.2. Contents and Conditions of Experiment

Table 1 shows the conditions for the model experiment. As shown in Table 1, we performed 20 cases for the 1-layer model, and 20 cases for the 2-layer model experiment. The behavior of settlement was observed through an acrylic plate during the loading test. The tension force was equally applied on both sides by using the loading plates (see Figure 1), on which some weights (50 N/each weight) were placed. When the tension force was greater than 200 N, the extension of the geosynthetics occurred. That is why we selected the tension forces (50, 100, 150, and 200 N) in the test, as shown in Table 1.

**Figure 2:**
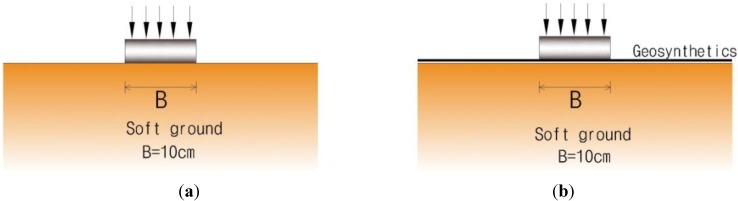

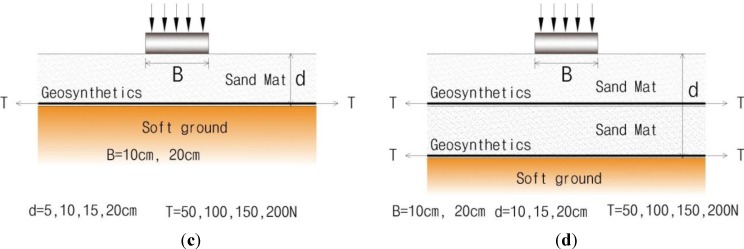
Schematic diagram of conditions for experiment. (**a**) unreinforced case; (**b**) geosynthetics only case; (**c**) 1 layer sand mat+geosynthetics case; (**d**) 2layer sand mat+geosynthetics case.

Figure 2 shows schematics for four different loading conditions, such as condition of loading on soft ground, condition of loading after laying geosynthetics on soft ground, condition of loading after laying sand-mat, and condition of loading after additional the laying of sand-mat, in two layers, to make a prescribed height of geosynthetics.

**Table 1:** Conditions for 1-layer and 2-layer sand-mat experiment.

Conditions for 1-layer sand-mat experiment					Conditions for 2-layer sand-mat experiment					
TEST. No.	Width of loading plate B (cm)	Thickness of sand layer (cm)	Tensile strength T (N)	Existence of reinforcement material	TEST. No.	Width of loading plate B (cm)	Thickness of 1-layerd/2 (cm)	Thickness of 2-layer d/2 (cm)	Tensile strength T (N)	Existence of reinforcement material
1N1	10	0	0	×	2N1	10	5	5	0	○
1N2	10	0	0	○	2N2	10	5	5	50	○
1N3	10	0	50	○	2N3	10	7.5	7.5	0	○
1N4	10	0	100	○	2N4	10	7.5	7.5	50	○
1N5	10	0	150	○	2N5	10	10	10	0	○
1N6	10	0	200	○	2N6	10	10	10	50	○
1N7	10	5	0	○	2N7	20	5	5	0	○
1N8	10	5	100	○	2N8	20	7.5	7.5	0	○
1N9	10	10	100	○	2N9	20	10	10	0	○
1N10	10	15	100	○	2N10	20	7.5	7.5	50	○
1N11	10	5	200	○	2N11	20	7.5	7.5	100	○
1N12	10	10	200	○	2N12	20	7.5	7.5	150	○
1N13	10	15	200	○						
1N14	20	0	0	×						
1N15	20	0	0	○						
1N16	20	0	100	○						
1N17	20	5	100	○						
1N18	20	10	100	○						
1N19	20	15	100	○						
1N20	20	20	0	○						

Note: × not used; and ○ used.

## 3. Results of Experiment and Analysis of Laboratory Model Experiment

### 3.1. Assumption of Ultimate Bearing Capacity

When a loading test is conducted for soft conditions, local shear failure or intrusion failure occurs in general. However, if geosynthetics and a sand layer are laid at the upper part of the soft ground, the form of failure becomes significantly different, which shows the trend of continuous increase in bearing capacity against settlement. In this case, we are unable to obtain ultimate bearing capacity, as we cannot find the peak point. Accordingly, in this research, we decided to analyze the result of the experiment, based on assumption that ultimate bearing capacity is the bearing capacity at the time when the width of the loading plate settled by 30%. Namely, in the case that the size of the loading plate is 10 cm under the condition of *q*_u_ = *q*_0.3_, the bearing capacity at the time when the settlement of 3 cm occurred was called ultimate bearing capacity *q*_u_, while, in the case of a loading plate of 20 cm, the bearing capacity at the time of settlement of 6 cm was called ultimate bearing capacity *q*_u_.

According to the research conducted by Terzaghi [16], the ultimate bearing capacity of shallow foundation is generated in the range of 4%–10% of settlement against the width of the foundation. Such a condition can be established when shear failure occurs at the first part of the ground. He also mentioned that, in case local shear failure or intrusion shear failure occur at the continuous foundation or strip foundation, ultimate bearing capacity is generated in the range of 15%–25% of settlement against the width of the foundation. In general, the settlement quantity of shallow foundation at a site is to be generated within the range of settlement quantity mentioned above.

Accordingly, in this research, settlement quantity is generated at 30% of the width of the foundation and the time of selection of ultimate bearing capacity was made based on the assumption that the foundation is in the form of continuous foundation, where plane strain occurs and, also, that the bearing capacity of foundation ground is induced significantly less than an actual site, as the foundation ground is for the purpose of a laboratory experiment. To be conservative, ultimate bearing capacity was determined to use the settlement quantity at the time when it is 30% of the width of the loading plate in order to clearly identify the reinforcement effects of geosynthetics and sand-mat.

### 3.2. The Case of 1-Layer Structured Sand-Mat Test

#### 3.2.1. Influence of Tensile Strength Applied to Geosynthetics on Bearing Capacity

**Figure 3:**
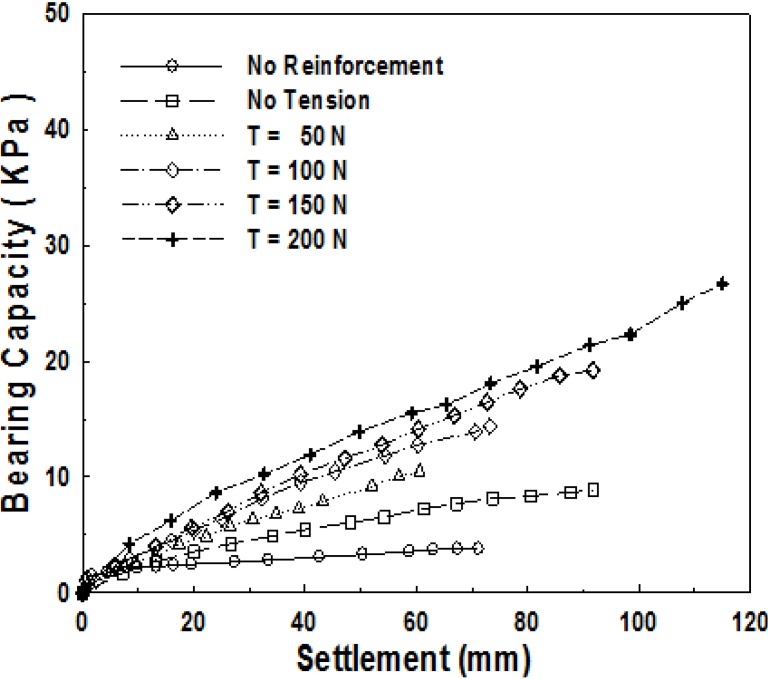
Relationship between bearing capacity and settlement for tensile load of ultimate bearing capacity depending on the level of settlement.

We can presume that tensile strength is being applied to surrounding geosynthetics, laid on soft ground, at an actual site in case local load is applied to the ground due to the contact force of geosynthetics, based on cohesion of soft ground. Accordingly, in the laboratory model experiment, it was arranged in such a way that tensile strength is applied to geosynthetics by applying an artificial load to geosynthetics. Figure 3 shows the relationship between bearing capacity curves and settlements for five kinds of tensile strengths, 0, 50, 100, 150, and 200 N. For comparison, the results of experiments on the ground, where geosynthetics were not used, are also presented.

Here, a tensile strength of 50 N means the tensile strength applied to geosynthetics from one side, which means respective loads applied to the width of geosynthetics, of 0.3 m, from both sides. It is converted into the tensile strength applied, per unit length, of approximately 340 N/m. Here, the value of 340 N/m is very small as compared to a maximum tensile strength of 50 kN/m for the reinforcement material used. On an actual site, however, it is possible for us to predict that very large tensile strength will be applied to geosynthetics. Figure 3 indicates that bearing capacity gets larger as tensile strength applied gets larger. The case of no reinforcement shows a very small ultimate bearing capacity at the level of 2 kPa, and the case of *T* = 200 N, with the assumption of *q*_u_ = *q*_0.3_, showed the bearing capacity of 10 kPa, which is about five times of that of the no-reinforcement case.

**Figure 4:**
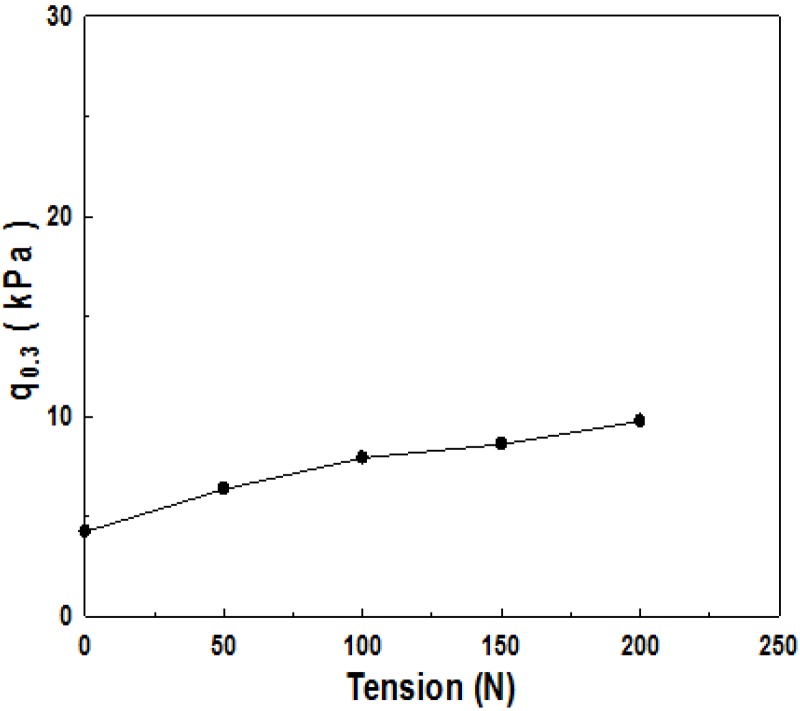
Change in bearing capacity with tensile load.

Figure 4 shows ultimate bearing capacity, depending on the size of the tensile strength applied to reinforcement material, which indicates that ultimate bearing capacity becomes larger as tensile strength gets larger.

#### 3.2.2. Influence of the Thickness of Sand-Mat Layer

It is very important to determine the appropriate thickness of sand-mat in actual design. Excessive thickness, over an appropriate level, may be safer, but it will be very expensive.

**Figure 5:**
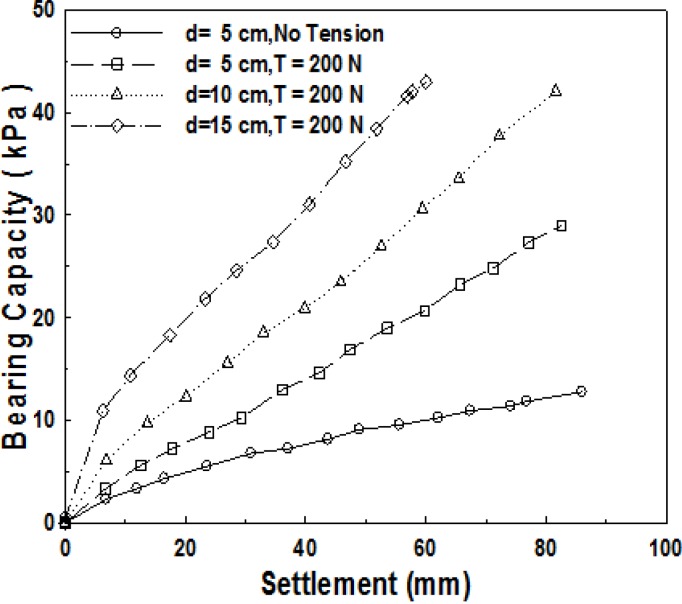
Bearing capacity with increasing thickness of sand-mat.

**Figure 6:**
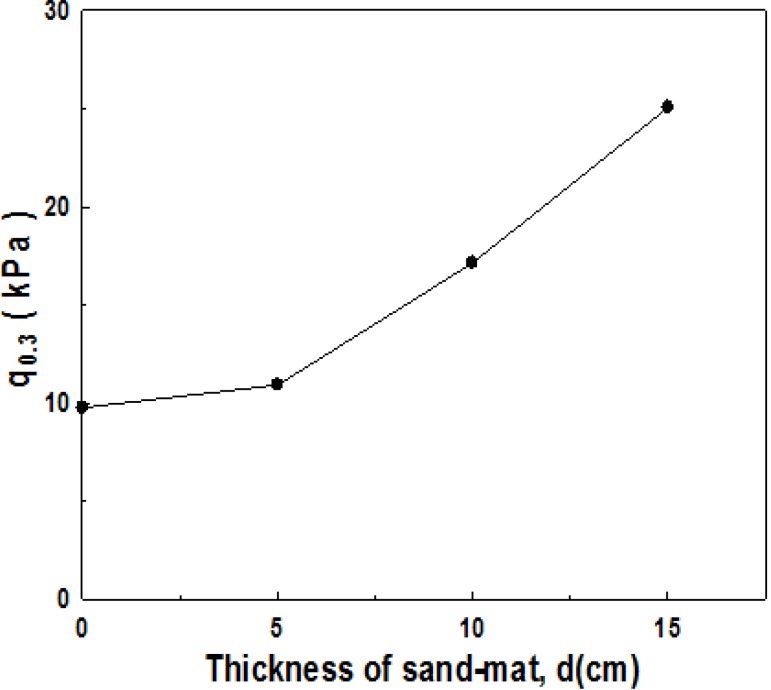
Bearing capacity with the change in the thickness of sand-mat (*q*_0.3_).

In order to identify the influence of the thickness of sand-mat on bearing capacity, model experiments were carried out for different thicknesses of sand-mat. Figure 5 shows the comparison of the results of four kinds of experiments using settlement-bearing capacity curves. Three cases are for the where the thicknesses of the sand-mats are 5, 10, and 15 cm, respectively, and the applied tensile strength was 200 N for all the three cases. The remaining case is for where the depth of the sand-mat is 5 cm, when the applied tensile strength is 0. Figure 5 shows that the bearing capacity gets far larger as the thickness of sand-mat increases. This means that increase in the thickness dispersion of load gets larger due to the sand-mat, which eventually results in the increased effect of bearing capacity of ground. Figure 6 indicates the ultimate bearing capacity obtained, assuming that the ultimate bearing capacity is the bearing capacity when the settlement quantity is 30%, and shows the ultimate bearing capacity depending on the thickness of sand-mat. In Figure 6, we can see a linear increase of ultimate bearing capacity with increasing thickness.

### 3.3. The Case of 2-Layer Structured Sand-Mat Test

#### 3.3.1. Influence of Tensile Strength Applied to Geosynthetics on Bearing Capacity

**Figure 7:**
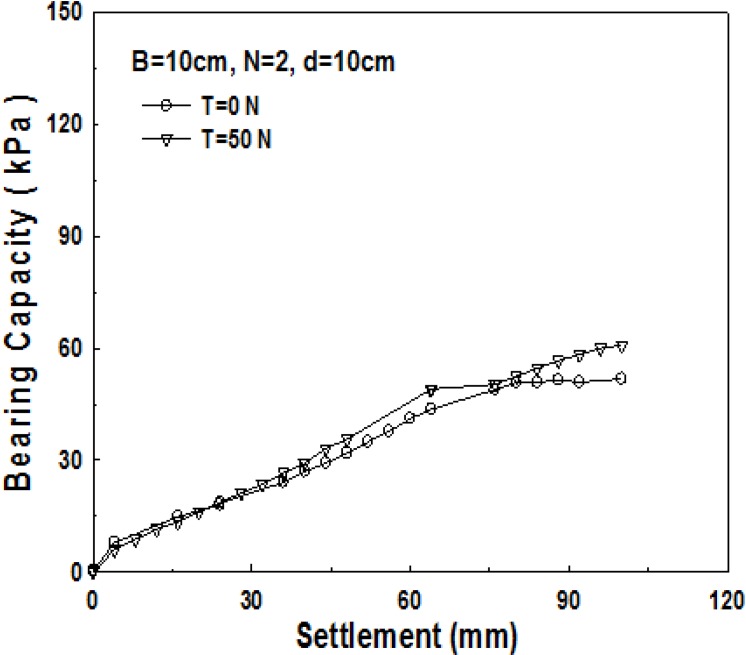
Change in bearing capacity with the tensile loads (*B* = 10 cm, *d* = 10 cm, *N* = 2).

**Figure 8:**
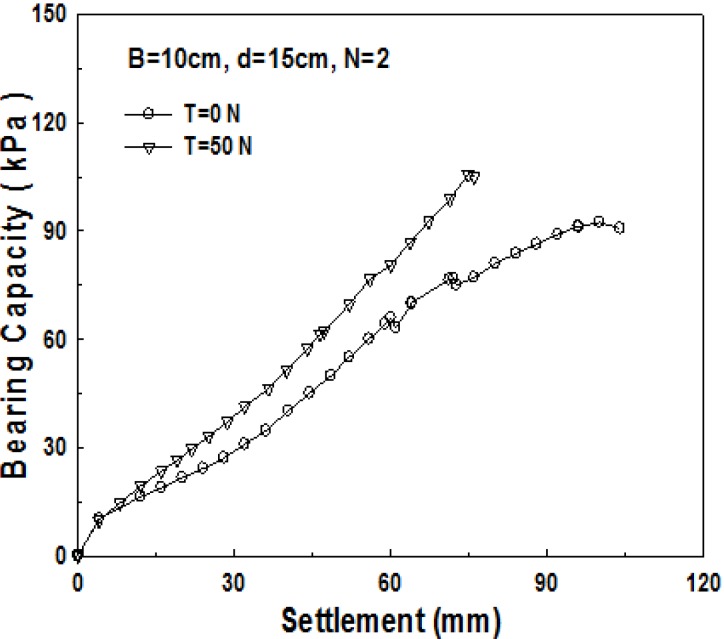
Change in bearing capacity with the tensile loads (*B* = 10 cm, *d* = 20 cm, *N* = 2).

**Figure 9:**
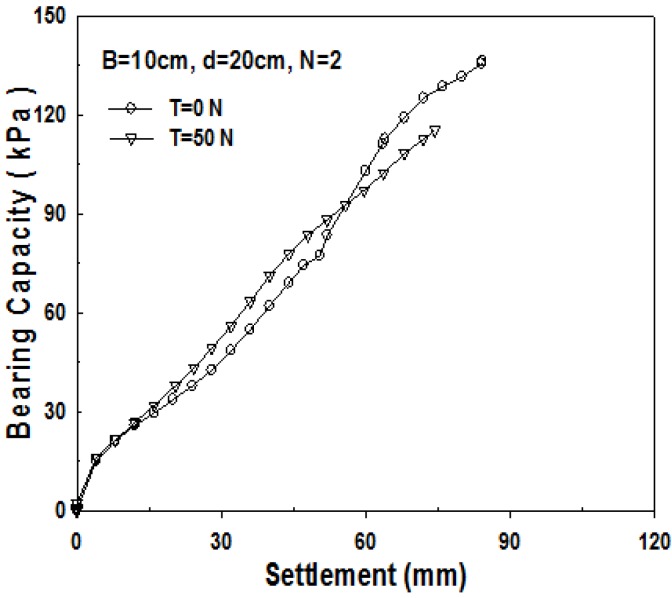
Change in bearing capacity pursuant to load (*B* = 10 cm, *d* = 20 cm, *N* = 2).

As geosynthetics are laid in two layers in the 2-layer structured sand-mat, two devices, which lift up the pendulum, are required in the apparatus for the experiment. That is to say, a loading test is carried out after applying tensile strengths of the same size to the geosynthetics in the upper part, and the geosynthetics in the lower part as well. Figure 7, Figure 8 and Figure 9 show the relationship between bearing capacity and settlement in the case where tensile strength was not applied to geosynthetics in 2-layer structured sand-mat, and in case where a tensile strength of 50 N was applied.

Figure 8 shows the relationship between the bearing capacity and settlement for 10 cm thick sand-mat. As settlement increases, bearing capacity increases slightly, initially, then it becomes larger with increasing tensile strength. Figure 8 shows the bearing capacity *vs.* settlement for 15 cm thick sand-mat. It is found that, even from the beginning of settlement, the bearing capacity for this case is fairly large and the difference in bearing capacity between tensile strengths of 0 N and 50 N gradually gets larger.

**Figure 10:**
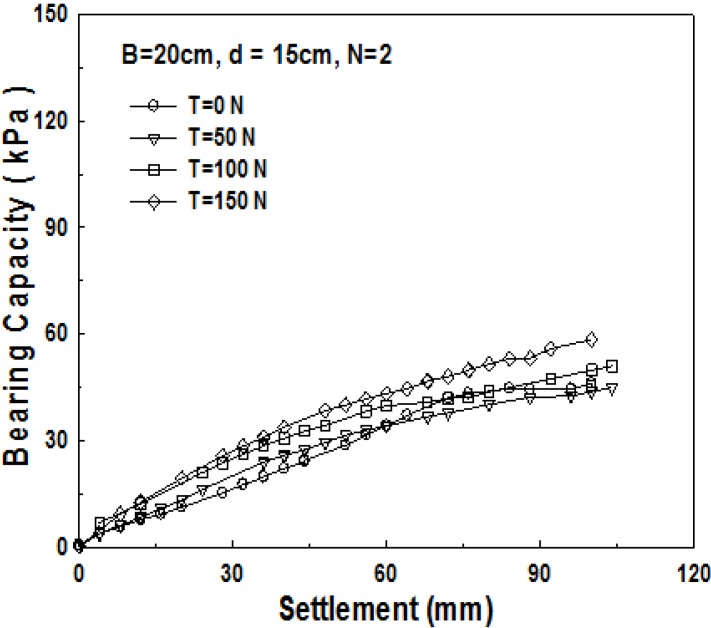
Relationship of bearing capacity and settlement pursuant to load (*B* = 20 cm, *d* = 15 cm, *N* = 2).

Figure 9 shows the relationship between the bearing capacity and settlement for 20 cm thick sand-mat. Up to 60 mm of settlement, the bearing capacity for the case where tensile strength is applied is larger, however, then, the bearing capacity for the case where tensile strength is not applied shows a larger value. The reason for this is because the tensile strength applied to geosynthetics cannot play its role due to excessively large settlement. Figure 10 shows the relationship between the bearing capacity and settlement for 15 cm thick sand-mat, with tensile strengths of 0, 50, 100, and 150 N. The results show that differences are not that large but bearing capacity becomes larger as the applied tensile strength gets larger. It is noted that, however, in the case of the applied tensile strength of 100 N, bearing capacity tends to decrease for settlement greater than 70 mm.

#### 3.3.2. Influence of Thickness d in the 2-Layer Structured Sand-Mat

**Figure 11:**
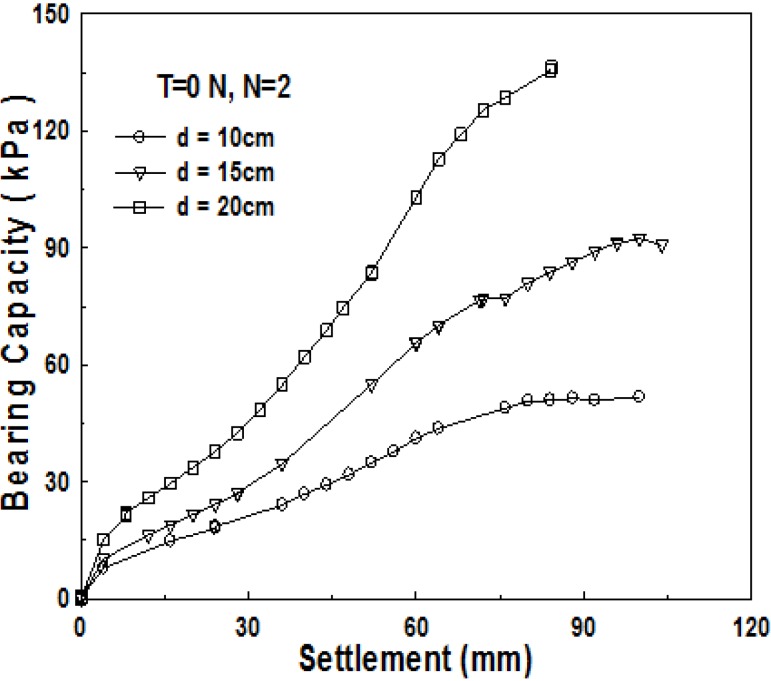
Relationship between bearing capacity and settlement for thickness of sand-mat (*B* = 20 cm, *N* = 2, *d* = 10 cm, 15 cm, 20 cm, T = 0 N).

**Figure 12:**
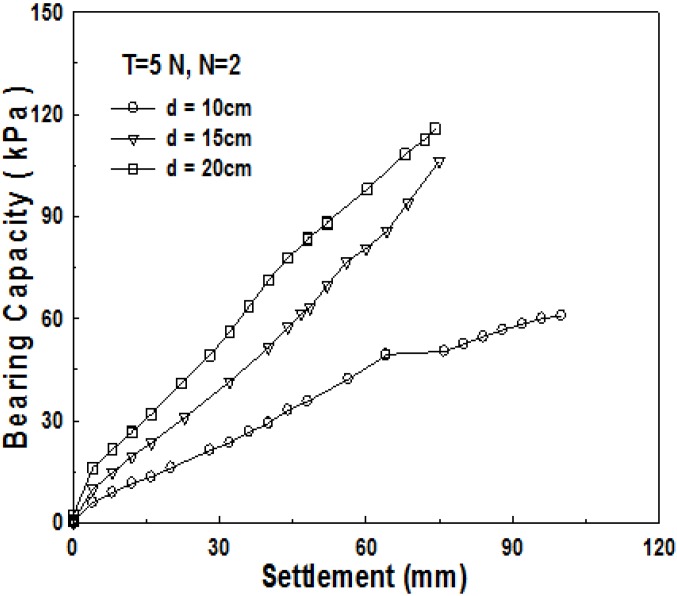
Relationship between bearing capacity and settlement for thickness of sand-mat (*B* = 20 cm, *N* = 2, *d* = 10, 15, 20 cm, *T* = 50 N).

As shown previously, the most influential factor for the increase of bearing capacity in the 1-layer structured sand-mat is the thickness of sand-mat. Figure 11 and Figure 12 show the result of the tests conducted for model ground in the 2-layer structured sand-mat, by changing the thickness of sand-mat upward to 10, 15, and 20 cm. As shown in Figure 11 and Figure 12, bearing capacity increases with an increase in settlement, showing a significantly large difference in bearing capacity as thickness *d* increases. It is found that, even in the 2-layer structured sand-mat, increasing the thickness of sand-mat layer is also the most effective method of obtaining a large bearing capacity.

### 3.4. Comparison of Bearing Capacities of 1-Layer Structured Sand-Mat and 2-Layer Structured Sand-Mat

**Figure 13:**
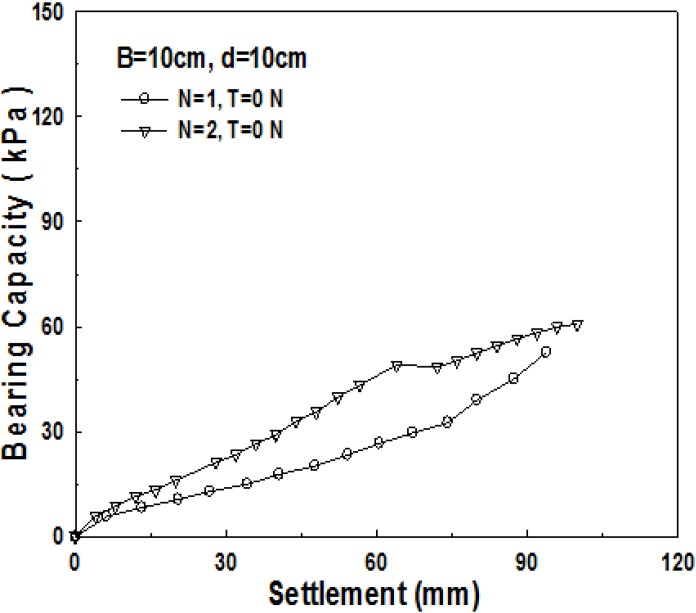
Relationship of bearing capacity and settlement pursuant to the number of reinforcement layers (*B* = 10 cm, *d* = 10 cm, *N* = 1, N = 2, *T* = 0 N).

**Figure 14:**
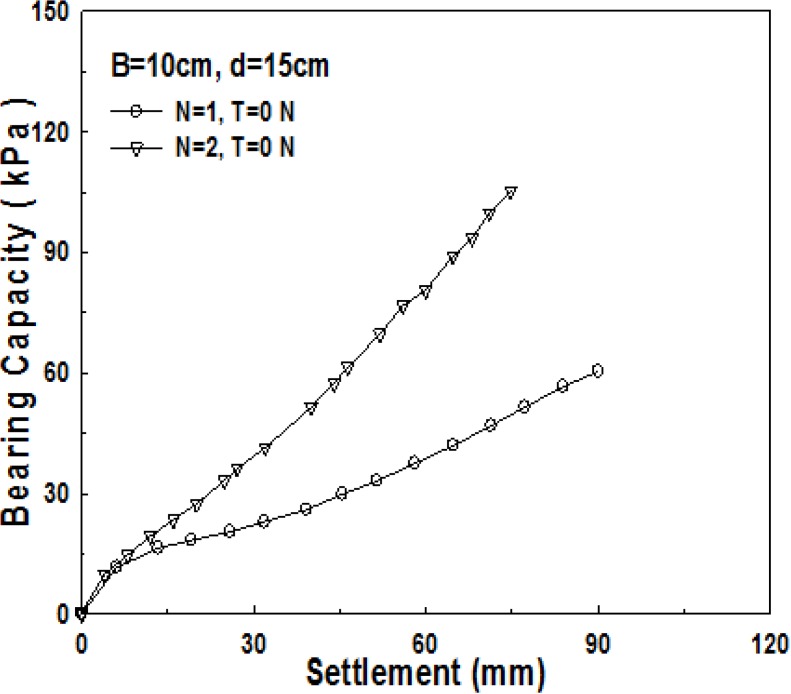
Relationship of bearing capacity and settlement pursuant to the number of reinforcement layers (*B* = 10 cm, *d* = 15 cm, *N* = 1, *N* = 2, *T* = 0 N).

**Figure 15:**
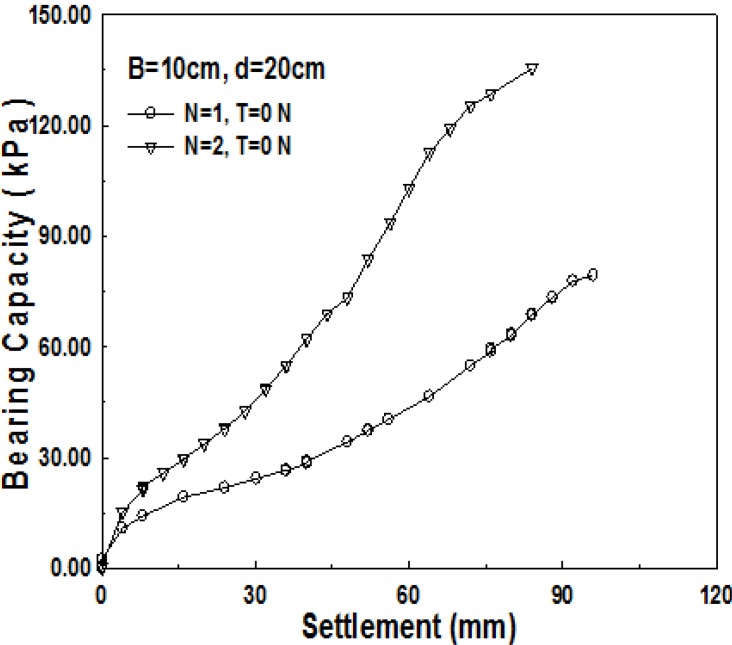
Relationship of bearing capacity and settlement pursuant to the number of reinforcement layers (*B* = 10 cm, *d* = 20 cm, *N* = 1, *N* = 2, *T* = 0 N).

Compared to 1-layer structured sand-mat, one more layer of geosynthetics is laid in the middle of a sand layer in the 2-layer structured sand-mat. Accordingly, it is common to assume that 2-layer structured sand-mat will have larger bearing capacity, even if the thicknesses are the same. Figure 13, Figure 14 and Figure 15 show the relationship of settlement and bearing capacity for 1-layer structured sand-mat and 2-layer structured sand-mat, for 10, 15, and 20 cm thickness, respectively. In the case of a thickness of 10 cm, the difference is not large, whereas the differences are large in the cases of 15 cm and 20 cm thicknesses.

Based on what is assumed as ultimate bearing capacity *q*_0.3_, the results of tests show a significantly large increase in bearing capacities of 75% and 90% in the cases of the thicknesses of 15 cm and 20 cm, whereas there is approximately a 34% increase in bearing capacity in the case of the thickness of 10 cm. This implies that the geosynthetics, laid in between the layers, played very significant role for the increase in bearing capacity.

**Figure 16:**
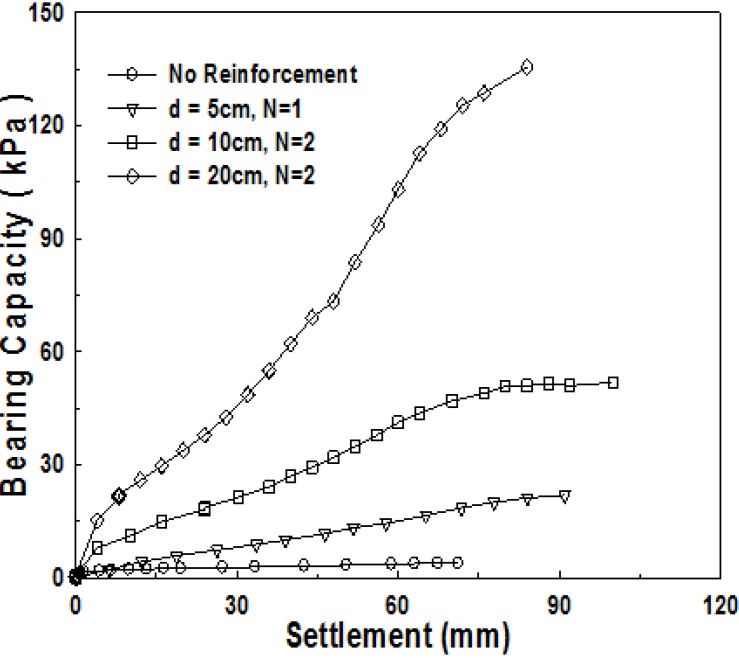
Relationship of bearing capacity and settlement pursuant to the number of reinforcement layers (*B* = 10 cm, *N* = 1 (*d* = 5 cm), *N* = 2 (*d* = 10 cm, *d* = 20 cm), *T* = 0 N).

Figure 16 shows the comparison of the test results for four cases of ground with no reinforcement, 1-layer structured sand-mat, with the thickness of 5 cm, and 2-layer structured sand-mat, with the thicknesses of 10 cm and 20 cm. The test results reveal that the bearing capacities of 1-layer structure and 2-layer structure are significantly larger when compared to no reinforcement. Two-layer structure shows a significantly larger bearing capacity when compared to 1-layer structure. The results reveal the effect of increasing bearing capacity to approximately 200% to 500%.

## 4. Design of Sand-Mat System

### 4.1. Concept of Bearing Capacity of Sand-Mat

**Figure 17:**
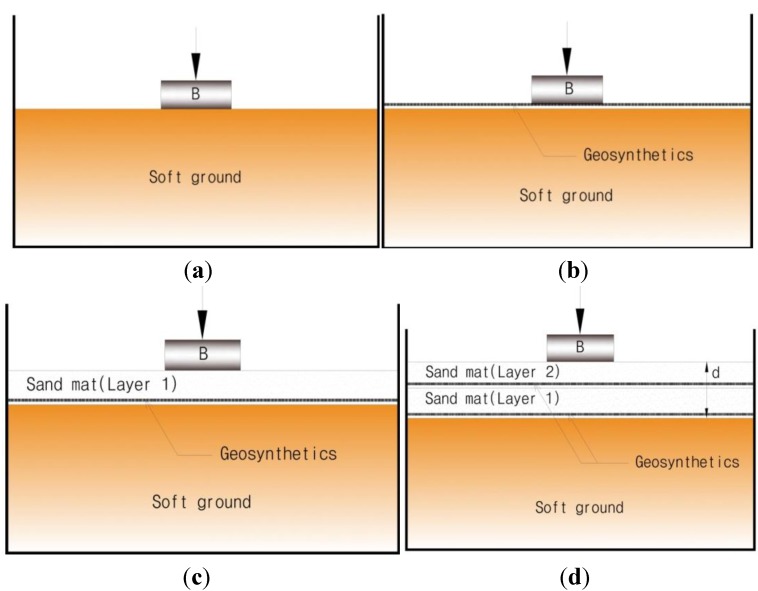
Bearing capacity of sand-mat system with considering various ground conditions. (**a**) ultimate bearing capacity of soft ground (*q*_u1_); (**b**) ultimate bearing capacity of geosynthetics reinforcement (*q*_u2_); (**c**) ultimate bearing capacity of 1-layer structured sand-mat (*q*_u3_); (**d**) ultimate bearing capacity of 2-layer structured sand-mat (*q*_u4_).

Bearing capacity of sand-mat can be categorized by construction stage, as shown in Figure 17. Namely, bearing capacities are categorized into ultimate bearing capacity of soft ground (*q*_u1_), ultimate bearing capacity of geosynthetic reinforcement for the bottom of foundation (*q*_u2_), ultimate bearing capacity of 1-layer structured sand-mat (*q*_u3_), and ultimate bearing capacity of 2-layer structured sand-mat (*q*_u4_). The above bearing capacities will have the relationships of *q*_u1 __< _*q*_u2 __< _*q*_u3 __< _*q*_u4_.

**Figure 18:**
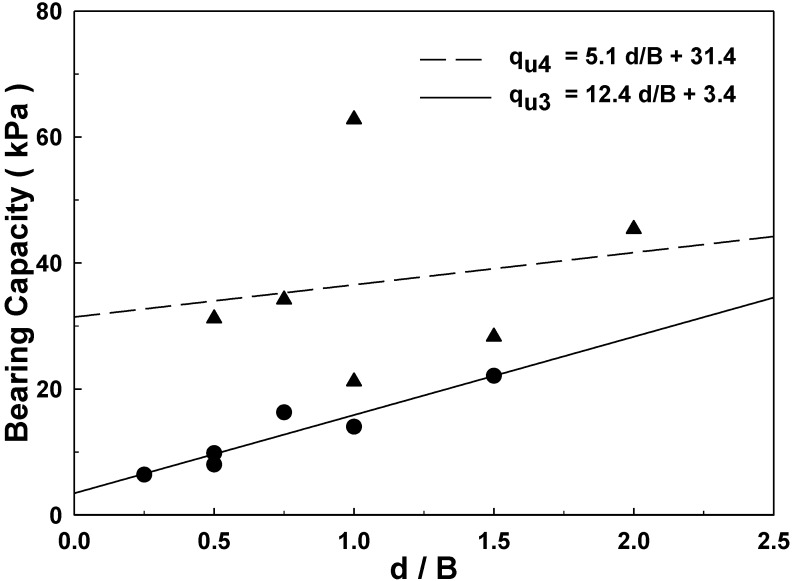
Regression analysis of 1-layer and 2-layer sand-mat system for ultimate bearing capacity.

As shown in Figure 17, the ultimate bearing capacity of 1-layer structured sand-mat can be expressed as the following, Equation (1):

*q*_u3_ = *q*_u2_ + (*q*_u3_ − *q*_u2_) (1)

where (*q*_u3_ − *q*_u2_) is the bearing capacity to be added by laying a sand layer. The ultimate bearing capacity of 2-layer structured sand-mat system can be expressed as the following Equation (2), as shown in Figure 18.

*q*_u4_ = *q*_u2_ + (*q*_u3_ − *q*_u2_) + (*q*_u4_ − *q*_u3_) (2)

where (*q*_u4_ − *q*_u3_) is the bearing capacity to be additionally generated by laying geosynthetics in the middle of a sand layer.

### 4.2. Analysis of Bearing Capacity with the Thickness of Sand-Mat

From the result of model experiments for 1-layer structured sand-mat system, the relationships of ultimate bearing capacities pursuant to d/B can be found using regression analysis, as shown in Figure 18, and the result of regression analysis is as the following Equation (3):

*q*_u3_ = 12.4 *d*/*B* + 3.4 (0.5 ≤ *d*/*B* ≤ 1.5) (3)

where *q*_u3_: Ultimate bearing capacity of a 1-layer structured sand-mat system; d/B: Value for which the thickness of the sand layer is divided by the width of loading plate B.

From the results of model experiments for a 2-layer structured sand-mat system, we identified the relationships of ultimate bearing capacities pursuant to *d*/*B* using regression analysis, as shown in Figure 18, and the result of regression analysis is as the following Equation (4):

*q*_u4_ = 5.1 *d*/*B* + 31.4 (0.5 ≤ *d*/*B* ≤ 2.0) (4)

where *q*_u4_: Ultimate bearing capacity of a 2-layer structured sand-mat system; *d*/*B*: Ratio of laying (Thickness of sand-mat laying/width of loading plate).

### 4.3. Bearing Capacity Increase Effect of Sand-Mat System

Among the methods of reinforcing soft ground, the sand-mat system is one of the most effective methods. Sand-mat is usually designed as a 1-layer structured system and is used in actual sites. Of note is that accurate concepts for design for the bearing capacity of 2-layer and multi-layer structured sand-mat systems have not been established yet. In this research, we obtained the rate of increase in bearing capacity, *i.e*., bearing capacity ratio (BCR) of each stage of the bearing capacity of original ground and used it for analysis. As explained earlier, the ultimate bearing capacity was assumed as the bearing capacity at the time when there is 30% settlement in the width of the loading plate. Indicating the case of reinforcement with geosynthetics only as *BCR*_0_, the case of a 1-layer structure as *BCR*_layer1_, and the case of a 2-layer structure as *BCR*_layer2_, BCR’s are defined as the following equations:

(5) $$BCR_{0}=\frac{q_{u2}}{q_{u1}}$$

(6) $$BCR_{\mathrm{Layer}1}=\frac{q_{u3}}{q_{u1}}$$

(7) $$BCR_{\mathrm{Layer}2}=\frac{q_{u4}}{q_{u1}}$$

**Figure 19:**
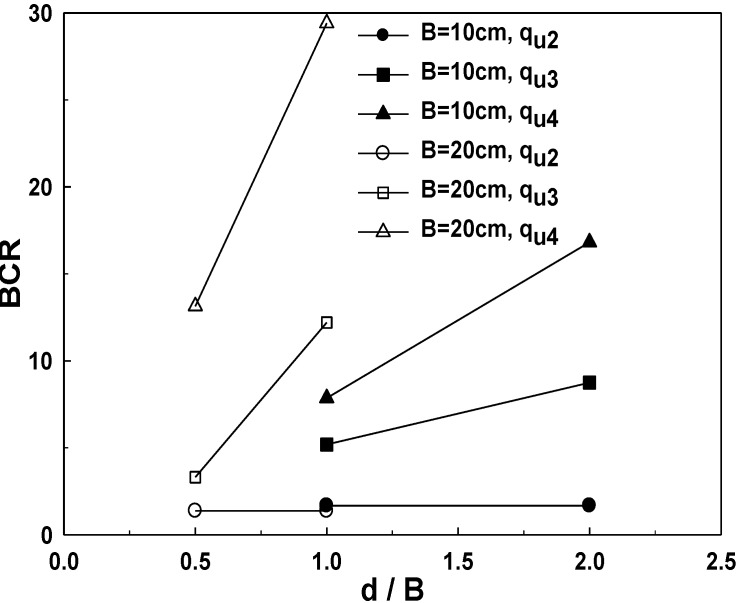
BCR pursuant to the ratio of the thickness of sand-mat (*d*/*B*) (*B* = 10 cm) and (*B* = 20 cm).

Figure 19 shows schematics of BCR with the width of the loading plate, and as shown in the Figures, BCR is significantly increased as the width of loading plate gets larger. BCR for 2-layer structure is increased by more than two times when compared to that of a 1-layer structure, and in the case of 2-layer reinforcement, bearing capacity is increased by up to 7.85–29.4 times when compared to that of no reinforcement.

### 4.4. Proposal for an Equation for Ultimate Bearing Capacity Using the Concept of Dispersion Angle

#### 4.4.1. Modification of Yamanouchi Equation

In the case of a 1-layer structured sand-mat system, actual design for a sand-mat layer has been generally been done using the following equation for ultimate bearing capacity, proposed by Yamanouchi [1].

(8) $$q_{u}=\left(1+\frac{d-D_{f}}{B}\right)\times \left[5.3C+T_{a}\left(\frac{2{\text{sin}\theta }_{2}}{B}+\frac{1}{r}\right)+\frac{4S_{a}R\left(1-{\text{cos}\theta }_{1}\right)}{B+d-D_{f}}+{\text{γ}}_{1}D_{f}\right]$$

where *q*_u_ ultimate capacity, *d* thickness of sand layer; *D*_f_ settlement at the time of ultimate bearing capacity; *B* width of loading plate; *C* cohesion; *T*_a_ tension force of geosynthetics; θ_1_ load dispersion angle; θ_2_ angle between tension force and horizontal line of the sand layer; *S*_a_ shear resistance force between soil and geosynthetics; *R* large radius of sand-mat system; *r* small radius of sand-mat system; and γ_1_ unit weight of soil.

In the above equation, however, load dispersion angle is fixed as 26.57° and, therefore, we cannot obtain accurate ultimate bearing capacity using the equation. Accordingly, we can modify the equation as the following, Equation (8), by introducing the concept of load dispersion angle to Equation (7).

(9) $$q_{u}=\left(1+\frac{2d{\text{tan}\theta }_{1}-D_{f}}{B}\right)\times \left[5.3C+T_{a}\left(\frac{2{\text{sin}\theta }_{2}}{B}+\frac{1}{r}\right)+\frac{4S_{a}R\left(1-{\text{cos}\theta }_{1}\right)}{B+2d{\text{tan}\theta }_{1}-D_{f}}+\gamma_{1}D_{f}\right]$$

#### 4.4.2. Calculation Method and Comparison of Load Dispersion Angles

In order to obtain the values of dispersion angles for sand-mat structure from the results of the experiments, calculations should be done using the assumption that bearing capacity at the time of settlement ratio of *s*/*B* = 0.3, *q*_0.3_ is the ultimate bearing capacity, *q*_u_. Indicating the bearing capacity in the case of 0 thickness of a sand-mat layer, even if geosynthetics are laid, as *q*_u-1_, and the bearing capacity in the case of changing thicknesses of sand-mat layers as *q*_u_, under the assumption that load dispersion is done 2:1 in the case where a sand-mat layer with the depth of d exists, we obtain a balance equation like the following Equation (9), and the equation can be rearranged as the subsequent Equation (10).

(10) $$q_{u}B=q_{u-1}[B+\left(d-D_{f}\right]$$

(11) $$q_{u}=q_{u-1}\left(1+\frac{d-D_{f}}{B}\right)$$

However, actual sites do not show a 2:1 distribution, and the load dispersion angle will change depending on respective conditions. Accordingly, we can get the following Equation (11) when we substitute *d* with in θ_1_2dtanθ_1_ in Equation (10) and rearrange the equation:

(12) $$q_{u}=\left(1+\frac{2d{\text{tan}\theta }_{1}-D_{f}}{B}\right)q_{u-1}$$

From the above Equation (11), we can get the equation to get load dispersion angle θ_1_ as follows:

(13) $$\theta_{1}={\text{tan}}^{-1}\left[\frac{B}{2d}+\left(\frac{q_{u}}{q_{u-1}}-1\right)+\frac{D_{f}}{2d}\right]$$

where, *D*_f_ refers to the settlement at the time of ultimate bearing capacity. Accordingly, if we try to obtain the values for ultimate bearing capacities through experiments against respective conditions, we will be able to get the load dispersion capability of sand-mat structure.

**Figure 20:**
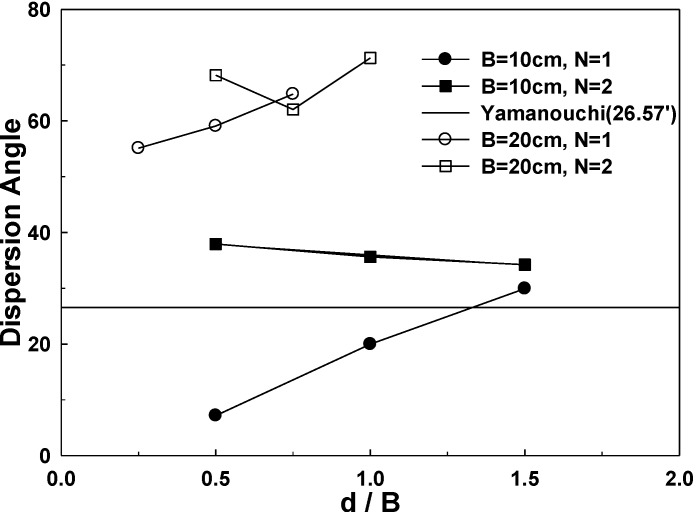
Dispersion angle of the 2-layer sand-mat by reinforced layers and laying ratio (*B* = 10 cm) and (*B* = 20 cm).

We analyzed and reviewed the load dispersion angles for 1-layer structured sand-mat systems and 2-layer structured sand-mat systems, respectively, using thickness and the size of loading plate. Now, we are going to compare 1-layer structure and 2-layer structure, simultaneously. Figure 20 shows the dispersion angles of 1-layer structure and 2-layer structure in the case of the widths of loading plates of 10 cm and 20 cm

First of all, in Figure 18, where the width of loading plate is 10 cm, dispersion angles of 26.57° tend to be gradually getting bigger, depending on the thickness in the case of a 1-layer structure, whereas the dispersion angles are, rather, smaller than in case the thickness of sand layer is small, such as 5 cm and 10 cm. However, 2-layer structure shows the characteristics of increasing in thickness while showing slightly decreasing dispersion angles, but the dispersion angle is larger than 30°, which is larger than 26.57°, which is applied to the Yamanouchi equation.

In Figure 20, where the width of loading plate is 20 cm, dispersion angles tend to be significantly larger, depending on the thickness in the case of 1-layer structure, and dispersion angle has significantly large values of, approximately, 40°–55°. In the case of a 2-layer structure, however, dispersion angles are initially decreased and increased later as the thickness is increased but dispersion angles are far larger when compared to those of a 1-layer structure, showing the distribution of 60°–75°.

This shows that dispersion angles of a 2-layer structure are far larger when compared to those of a 1-layer structure, which implies that the adoption of 2-layer reinforcement in the design of sand-mat layers will generate the effect of a very large increase in bearing capacity.

#### 4.4.3. New Equation for Bearing Capacity for Load Dispersion Angle

When we simplify the above Equation (8) by eliminating the items which generate minute values, we can get the following Equation (13):

(14) $$q_{u}=\left(1+\frac{2d{\text{tan}\theta }_{1}-D_{f}}{B}\right)\times \left[5.3C+T_{a}\left(\frac{2{\text{sin}\theta }_{2}}{B}+\frac{1}{r}\right)+{\text{γ}}_{1}D_{f}\right]$$

When we do calculations, applying usual average value of 30° for θ_2_ for a safety side design, neglecting item *r* from the above equation, we can get the following Equation (14). When we eliminate all minute values from the original equation, the design can be regarded as a safety side design, even though we cannot calculate accurate ultimate bearing capacity.

(15) $$q_{u}=\left(1+\frac{2d{\text{tan}\theta }_{1}-D_{f}}{B}\right)\times \left[5.3C+\frac{T_{a}}{B}+{\text{γ}}_{1}D_{f}\right]$$

We can say that the above equation can be used for an actual design of a sand-mat system. Compared with existing equations, it is clear that the ultimate bearing capacity is calculated by substituting the dispersion angle suitable for the system instead of using a fixed dispersion angle. The above equation is a new equation for bearing capacity, and using the substitution of load dispersion angle the equation can be applied to a 2-layer structured sand-mat system as well. However, note that *T*_a_ is the allowable tensile strength to be applied to the lower part of geosynthetics, and it is desirable to use *T*_a_ for the strength of geosynthetics which are to be installed in the central area of a sand layer.

## 5. Summary and Conclusions

In this research, we conducted laboratory experiments for the calculation of appropriate bearing capacity to be applied to the design of a soft ground reinforcement method, using geosynthetics and sand-mat. Based on the results of analyses, the following conclusions can be drawn.

- The result of examination of bearing capacity ratio (BCR) pursuant to the structure of sand-mat reveals that BCR for 2-layer sand-mat is increased to more than two times that of 1-layer sand-mat and the bearing capacity of 2-layer reinforcement is increased up to 7.85–29.4 times that of no reinforcement.
- We proposed a new bearing capacity equation by modifying the equation proposed by Yamanouchi. We can get ultimate bearing capacities of 1-layer and 2-layer structured sand-mat using the following equation:
  (16) $$q_{u}=\left(1+\frac{2d{\text{tan}\theta }_{1}-D_{f}}{B}\right)\times \left[5.3C+\frac{T_{a}}{B}+{\text{γ}}_{1}D_{f}\right]$$
- The result of calculation of bearing capacities based on the proposed new equation shows large differences compared with the calculation based on Yamanouchi's equation, and the bearing capacities of 1-layer structured sand-mat systems and 2-layer structured sand-mat systems tend to show big differences depending on load dispersion angle. The bearing capacity of a 1-layer structured sand-mat system, calculated using the new equation, is smaller or increased to more than two times, respectively, depending on the width of the loading plate, but the bearing capacity of a 2-layer structured sand-mat system is significantly increased when compared with the result using the Yamanouchi equation, showing a significant increase of approximately 1.4–2.4 times, compared with a 1-layer structure.
- In case where the bearing capacity of a 1-layer structured sand-mat is not sufficient, or the thickness of the sand-mat layer is increased at the time of designing the sand-mat method as a countermeasure for soft ground, to secure bearing capacity, reinforcement by additional laying of geosynthetics within a sand layer, namely by making a 2-layer structure could increase bearing capacity significantly.
- In this research, we have studied the methods of increasing bearing capacity by reinforcing geosynthetics on soft ground, and by laying sand-mat, and the purpose of this research is to make a meaningful proposal for a new bearing capacity equation at the time of design for a geosynthetically-reinforced sand-mat method, based on the results of experiments. In the future, if quantitative values of load dispersion angles can be obtained through further research on the factors, which affect load dispersion angles, it will become an important factor in the design for the calculation of the bearing capacity of soft ground.
